# StealthX: A Versatile and Potent Exosome-Based Vaccine Platform for the Next Pandemic

**DOI:** 10.3390/vaccines13121239

**Published:** 2025-12-13

**Authors:** Minghao Sun, Kristi Elliott

**Affiliations:** Capricor, Inc., San Diego, CA 92121, USA

**Keywords:** StealthX, exosome, nanogram, multiplex

## Abstract

Exosome-based vaccines represent a transformative platform in modern vaccinology, combining nanoscale delivery, biocompatibility, and potent immunogenicity. Among these, the StealthX platform developed by Capricor, Inc. has demonstrated exceptional versatility, enabling antigen presentation at nanogram level doses without the need for adjuvants. Preclinical studies using StealthX have shown strong humoral and cellular immune responses against SARS-CoV-2 variants, including Delta and Omicron, as well as broader applications against influenza and RSV antigens. The platform’s ability to accommodate multiple antigens within a single formulation addresses the challenges of viral variation and facilitates multivalent “mix-and-match” immunization strategies. This review offers an in-depth evaluation of the StealthX vaccine platform, covering the biological mechanisms underlying exosome function, the engineering approaches used to load antigens, and preclinical results demonstrated across three pivotal studies. By synthesizing current evidence, this review underscores the platform’s applicability for emerging infectious diseases and explores the strategic value of multivalent exosome-based vaccines in global immunization efforts as an emerging next-generation vaccine technology.

## 1. Introduction

The COVID-19 pandemic has highlighted both the strengths and limitations of modern vaccine platforms, revealing an urgent need for more adaptable, effective, and scalable approaches [1]. Traditional vaccines such as live attenuated, inactivated, and subunit protein vaccines have been highly successful in preventing many infectious diseases over the last century [2]. However, these approaches often face challenges related to production timelines, cold-chain storage requirements, reactogenicity, and limitations in providing broad immunity against rapidly evolving pathogens [3]. For example, seasonal influenza vaccines require frequent reformulation due to antigenic drift, and some viral vector vaccines can be limited by preexisting immunity to the vector itself, reducing efficacy [4,5].

The advent of mRNA vaccines, particularly those delivered via lipid nanoparticles (LNPs), represented a paradigm shift in vaccinology, enabling rapid design, high efficacy, and flexible adaptation to emerging viral variants [6]. However, mRNA-LNP vaccines still present logistical and immunological challenges. They require ultra-cold storage to maintain stability, which complicates distribution, especially in resource-limited settings [7]. Additionally, while mRNA vaccines elicit strong antibody responses, their durability and breadth of protection against viral variants can be limited, often necessitating booster doses [8]. Moreover, systemic reactogenicity and rare adverse events have prompted careful monitoring and highlighted the need for alternative delivery platforms that balance potency, safety, and convenience [9].

Exosome-based vaccines have emerged as a promising alternative that may overcome many of these limitations [10]. Exosomes are nanoscale extracellular vesicles (~50–150 nm) secreted by virtually all cell types [11,12], involved in intercellular communication through the transfer of proteins, lipids, and nucleic acids [11]. Their lipid bilayer membrane and inherent natural uptake capabilities by cells allow for efficient delivery of biologically active cargo to recipient cells [13]. Importantly, exosomes are naturally biocompatible, generally none or low immunogenic, and capable of protecting enclosed molecules from degradation in circulation [13]. These properties make them uniquely suitable for vaccine applications, allowing for the presentation of antigens to the immune system in a manner that closely mimics natural infection maintaining natural presentation of viral antigens while avoiding the risks associated with live-attenuated or viral vector vaccines [14].

The StealthX platform exemplifies the potential of exosome-based vaccines. Engineered to display viral antigens in its natural conformation with none or minimum modification such as SARS-CoV-2 spike and nucleocapsid proteins on their surface [15]. Intriguingly, only nanogram-level doses delivered by StealthX exosomes is needed for effective immunization without requiring any additional adjuvants as most protein-based vaccines do [15]. Preclinical studies have demonstrated robust humoral immune responses, characterized by high titers of neutralizing antibodies, as well as strong cellular immunity, including antigen-specific CD4+ and CD8+ T-cell activation [15]. Importantly, these responses were observed against multiple SARS-CoV-2 variants, including Delta and Omicron, highlighting the platform’s potential to provide cross-protective immunity against rapidly evolving viruses [16].

Another significant advantage of StealthX is its flexibility for multivalent vaccine formulations [17]. By incorporating multiple antigens into a single exosome preparation, StealthX enables “mix-and-match” immunization strategies that can broaden the immune response and reduce the risk of viral escape [17]. This approach has been evaluated not only for SARS-CoV-2 but also for other viral pathogens, including influenza virus and respiratory syncytial virus (RSV), demonstrating the platform’s versatility and potential for rapid adaptation in response to emerging infectious diseases [17]. Additionally, the exosome delivery system ensures efficient antigen presentation while minimizing reactogenicity, a common limitation of conventional adjuvant-based protein vaccines [18].

In summary, the StealthX exosome-based vaccine platform represents a significant advancement in the field of vaccinology. By combining natural biocompatibility, efficient antigen delivery, potent immunogenicity, and multivalent capability, StealthX offers a versatile and effective approach to modern vaccine challenges. This review aims to synthesize current knowledge on the StealthX platform, integrating findings from three pivotal studies that investigate its design, immunogenicity, multivalent capabilities, and preclinical safety [15,16,17] We discuss mechanistic insights into exosome-based antigen delivery, compare StealthX to existing vaccine technologies, and highlight considerations for clinical translation. By providing a detailed evaluation of the platform, we aim to illustrate its potential to address current and future challenges in vaccine development, including rapid response to viral variants, broad immune coverage, and improved accessibility. The following sections of this review will explore the mechanistic basis of exosome vaccines, detailed engineering strategies employed in StealthX, preclinical immunogenicity and efficacy data, and considerations for future clinical application.

## 2. Exosome Biology and Vaccine Mechanisms

### 2.1. Overview of Exosomes

Exosomes are a subset of extracellular vesicles (EVs), nanoscale lipid bilayer particles typically ranging from ~50 to 200 nanometers in diameter [19]. They are secreted by nearly all mammalian cell types and play crucial roles in intercellular communication by transferring proteins, lipids, and nucleic acids, including mRNA and microRNA [11,12,20]. Biogenesis of exosomes begins in the endosomal system: early endosomes mature into late endosomes or multivesicular bodies (MVBs) containing intraluminal vesicles, which are released as exosomes upon fusion with the plasma membrane [21,22]. Their cargo reflects the cellular origin and physiological state, making them ideal carriers of biological information.

The natural role of exosomes in immunity includes antigen presentation, modulation of inflammatory responses, and activation of adaptive immune responses [14,23]. Dendritic-cell-derived exosomes, for instance, carry MHC–peptide complexes capable of stimulating T cells, highlighting the intrinsic potential of exosomes to function as vaccine delivery vehicles [24,25]. Moreover, exosomes possess inherent targeting properties; their surface molecules, such as tetraspanins (CD9, CD63, CD81), integrins, and adhesion proteins, facilitate uptake by specific recipient cells, enhancing the precision of antigen delivery [26]. In addition, with proper engineering, exosomes can be targeted to specific tissue or organs for targeted drug delivery [27,28].

### 2.2. Mechanistic Basis for Exosome-Based Vaccines

Exosome-based vaccines leverage the natural antigen-presenting capabilities of these vesicles [29,30]. Antigens can be incorporated into exosomes either by endogenous expression (engineering donor cells to express antigen fused with exosomal membrane proteins) or by post-isolation loading (chemical or physical incorporation of proteins or nucleic acids into isolated vesicles) [31]. In StealthX, the antigen-loading strategy involves genetic fusion of viral antigens, such as the SARS-CoV-2 spike or RSV fusion protein, with exosomal membrane anchoring domains to ensure surface display, facilitating direct interaction with B cells and professional antigen-presenting cells (APCs) [15]. In addition, with addition of signaling peptide, hinge domain and linker, it potentially could put any antigen presented on the surface of exosome.

Upon administration, exosome-based vaccines are internalized by APCs through endocytosis or direct membrane fusion. Biodistribution data showed that liver and spleen had highest distribution. Macrophages and dendritic cells naturally have highest cell uptake for exosome too [32]. The antigens are processed and presented via MHC class I and II pathways, activating CD8+ cytotoxic T lymphocytes and CD4+ helper T cells, respectively [29]. This dual activation is critical for robust and long-lasting immunity, providing both neutralizing antibodies and cellular immunity against intracellular pathogens [33]. Importantly, exosomes can carry multiple antigens simultaneously, enabling multivalent immune responses, a feature that is particularly advantageous for rapidly evolving viruses or pathogens with multiple immunodominant epitopes. Preclinical data demonstrated that dosing mice with a cocktail of exosomes in nanogram dosage which carry SARS-CoV-2 Spike, Influenza H3 and RSV Fusion protein could induce robust humoral immunity against all three viruses [17].

Preclinical studies have consistently demonstrated that exosome vaccines induce potent adaptive immune responses. Humoral immunity is characterized by the generation of high titters of antigen-specific IgG antibodies capable of neutralizing viral entry [34]. Cellular immunity involves activation of CD8+ cytotoxic T lymphocytes capable of targeting infected cells and CD4+ helper T cells that orchestrate the immune response [35,36]. StealthX studies, for instance, report strong T-cell cytokine responses (e.g., IFNγ, TNFα, IL-2) and B-cell activation following administration of nanogram level doses of SARS-CoV-2 spike and nucleocapsid antigens [15].

### 2.3. Advantages over Conventional Vaccine Platforms

Compared with protein subunit vaccines, exosome-based vaccines offer superior delivery efficiency. Traditional protein vaccines often require microgram-to-milligram quantities of antigen along with adjuvants to elicit meaningful immune responses [18]. In contrast, StealthX demonstrates that only nanogram level doses delivered via exosomes are sufficient to generate strong humoral and cellular immunity [15,16]. The exosome membrane maintains antigens with their natural conformation, enhancing bioavailability and prolonging antigen exposure to the immune system.

Compared to mRNA-LNP vaccines, exosome-based platforms offer greater biocompatibility and reduced reactogenicity [10]. LNP formulations, while effective in delivering mRNA, can trigger systemic inflammatory responses and require strict cold-chain conditions for stability [8,37]. Exosomes, being naturally derived, are generally well-tolerated, stable under refrigeration, and capable of delivering both proteins and nucleic acids without additional synthetic carriers [13]. Most importantly, mRNA-LNP vaccines had limitation for multiplexing due to toxicity induced by both mRNA and LNP [8]. On the contrary, exosome is the natural delivery system in the human body and has great safety profile and our data consistently demonstrated that only nanogram of antigen is needed for efficient immunization [16]. All of these granted great capability for StealthX exosome platform for multiple antigens administration simultaneously.

Compared with viral vector vaccines, such as adenovirus-based platforms, face limitations including preexisting immunity to the vector, which can reduce vaccine efficacy [5,38]. Exosomes, being cell-derived and lacking replicative capacity, circumvent these limitations. Furthermore, exosomes can be engineered to display antigens from multiple pathogens simultaneously, enabling multivalent immunization strategies without the risk of vector interference.

### 2.4. Multivalent Delivery and “Mix-and-Match” Potential

A unique advantage of exosome-based vaccines is their capacity to carry multiple antigens simultaneously. StealthX has demonstrated the feasibility of combining spike and nucleocapsid proteins within the same exosome, eliciting robust immune responses against both antigens without evidence of immune interference [15]. This multivalent capability facilitates “mix-and-match” strategies, allowing vaccines to target multiple viral strains or distinct pathogens within a single formulation [17]. This approach can accelerate responses to emerging infectious diseases and reduce the number of injections required, improving compliance and coverage.

## 3. StealthX Platform: Design and Engineering

### 3.1. Overview of the StealthX Platform

The StealthX platform represents an innovative approach to exosome-based vaccine delivery. It is designed to present viral antigens on the surface of exosomes produced by genetically engineered donor cells with their natural conformation. By leveraging the natural biocompatibility, stability, and targeting capabilities of exosomes, StealthX aims to achieve potent immunogenicity at remarkably low antigen doses (nanogram dosage) [16]. Unlike conventional vaccines that rely on microgram-to-milligram quantities of antigen, StealthX demonstrates that nanogram-level doses are sufficient to induce robust humoral and cellular immune responses, reducing material requirements and potential side effects [15]. Another striking point for StealthX vaccine is that it would not require addition of any adjuvant, which dramatically enhanced StealthX vaccine’s safety profile [18].

**Figure 1:**
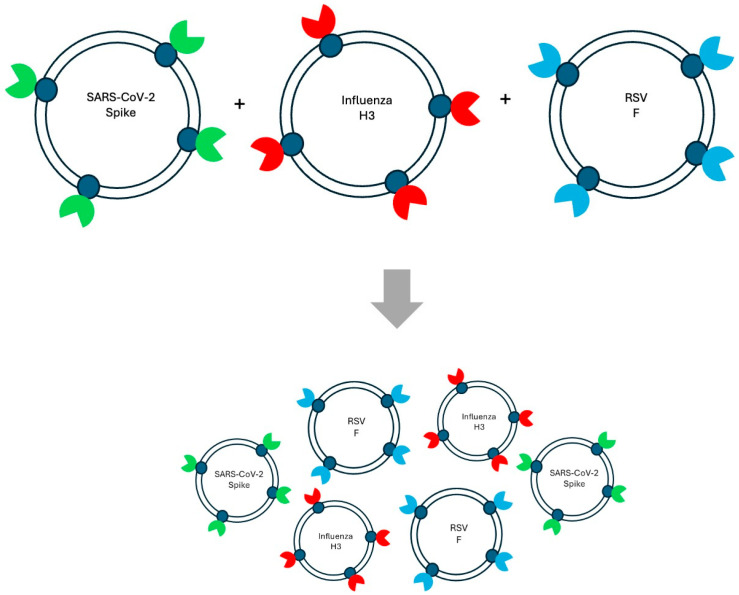
Schematic representation of Mix-and-Match strategy for multiplex vaccine.

The platform’s versatility lies in its capacity to accommodate multiple antigens simultaneously (Figure 1), enabling the development of multivalent vaccines. This capability is particularly valuable for pathogens with multiple immunodominant epitopes or for designing vaccines that target several viral variants in a single formulation. StealthX has been applied to antigens from SARS-CoV-2, influenza H3, and RSV, demonstrating broad applicability and adaptability [17].

### 3.2. Antigen Incorporation Strategies

The engineering of StealthX exosomes primarily involves genetic fusion of target antigens with exosome-associated membrane proteins to ensure surface display. Common fusion partners include tetraspanins such as CD63, CD81, CD9, or exosomal scaffolding proteins that localize antigens to the vesicle membrane [12,39]. This strategy allows the antigens to be presented in a conformation accessible to B-cell receptors and antigen-presenting cells, facilitating robust immune activation. For example, SARS-CoV-2 spike protein can be fused with the C-terminal domain of CD9 to anchor it onto the exosome surface, preserving its trimeric structure critical for neutralizing antibody recognition. Nucleocapsid proteins can also be incorporated in a similar manner with proper signal peptide and hinge domain, allowing simultaneous presentation of both antigens to the immune system, which is normally not presented on the surface of viral particles [15]. Preclinical studies have shown that surface display via exosomal scaffolds enhances antigen uptake by dendritic cells and promotes efficient T cell activation [24].

### 3.3. Multivalent and Mix-and-Match Design

A hallmark of StealthX is its multivalent capability, enabling display of multiple viral antigens in a cocktail of exosomes. Basically, multiple cells were engineered, and each cell expressed a different viral antigen. Each exosome was manufactured to only carry one viral antigen. At the end, different exosomes (Drug substance) were formulated to a single dose for multiple viruses. Instead of displaying multiple antigens in the same exosome, we could precisely control the dosage for each antigen. This approach addresses the challenge of viral variation and the need for broad immune coverage. In practice, donor cells can be engineered to express multiple antigen fusions simultaneously, resulting in exosomes that carry distinct viral proteins without evidence of immune interference. Studies have demonstrated the feasibility of combining SARS-CoV-2 spike and nucleocapsid antigens, eliciting both neutralizing antibodies against spike and T-cell responses against nucleocapsid [15]. Similarly, influenza H3 hemagglutinin and RSV fusion proteins have been incorporated in multivalent formulations together with SARS-CoV-2 spike protein, showing robust immunity against all three viruses in a single dose in preclinical models [17]. The “mix-and-match” strategy further allows combining different exosome preparations, each carrying a distinct antigen, offering flexibility in vaccine design and rapid adaptation to emerging variants.

### 3.4. Preclinical Characterization

StealthX exosomes undergo rigorous characterization to ensure consistency, stability, and functional integrity. Key assays include, but not limited to, nanoparticle tracking analysis, flowcytometry for exosome surface markers (CD63, CD81 and CD9), Western blot for contamination markers and cell type specific uptake assay by immunofluorescence [40,41,42]. In Table 1, common methods/assays for exosome characterization are summarized [40,41,42].

### 3.5. Manufacturing Considerations

The production of StealthX exosomes relies on scalable mammalian cell culture systems. Donor cells are genetically engineered to express the desired antigen fusions, and exosomes are harvested from conditioned media via ultracentrifugation, filtration, or tangential flow filtration [43,44]. Quality control includes assessment of exosome concentration, size distribution, purity (removal of cellular debris), and antigen incorporation. Batch-to-batch consistency is critical to ensure reproducible immune responses and regulatory compliance. Advances in exosome isolation and purification technologies are increasingly enabling large-scale production suitable for clinical trials and commercial deployment [45,46]. Moreover, the simplicity of exosome formulations—no requirement for adjuvants and compatibility with standard refrigeration—reduces manufacturing complexity and logistical hurdles compared with conventional protein or mRNA vaccines.

### 3.6. Advantages of StealthX Engineering

The design of StealthX confers multiple advantages over conventional vaccine platforms:

- Enhanced antigen presentation: Surface display ensures antigens are accessible to B cells and APCs, promoting both humoral and cellular responses.
- Reduced antigen dose: Nanogram-level dosing reduces production requirements and potential reactogenicity [15,16].
- Multivalent flexibility: Ability to carry multiple antigens facilitates broad immune coverage and rapid adaptation to emerging pathogens [17].
- Natural biocompatibility: Derived from mammalian cells, exosomes are inherently well-tolerated, reducing inflammatory responses compared with synthetic nanoparticles [13].
- Scalable and stable: Production in cell culture systems with standard storage requirements supports global deployment [45].

## 4. Immunogenicity and Preclinical Efficacy

### 4.1. Overview of Preclinical Models

To evaluate the immunogenicity and efficacy of the StealthX platform, multiple preclinical studies have employed small animal models, primarily mice and rabbits. These models allow detailed assessment of both humoral and cellular immune responses, as well as dose optimization and variant cross-reactivity. The three key studies forming the basis of this review examined StealthX vaccines delivering SARS-CoV-2 spike and nucleocapsid proteins, influenza H3 hemagglutinin, and RSV fusion proteins, either individually or in multivalent formulations [15,16,17]. Animal models were immunized via intramuscular injection, typically using nanogram-level doses of exosome-displayed antigens instead of using microgram-level as most protein based vaccine (>1000 fold compared with exosome based vaccine, StealthX) [15,16]. Booster doses were administered at intervals of 2–3 weeks in most protocols. Blood samples were collected at multiple time points to assess antibody titers and neutralization capacity, while splenocytes and lymph nodes were analyzed for T-cell activation and cytokine production. Comparative controls included equivalent doses of recombinant protein antigens without exosome delivery, highlighting the efficiency of the exosome platform. Due to no adjuvants used in our formulation, we did not detect any toxicity or adverse effect in all the test tissues including liver, kidney, spleen et al. [18].

### 4.2. Humoral Immune Responses

One of the most striking features of StealthX is its ability to induce strong humoral immunity with extremely low antigen doses (Nanogram dosage) [15,16]. In murine studies, nanogram quantities of SARS-CoV-2 spike protein delivered via StealthX exosomes elicited robust IgG antibody titers comparable to, or exceeding, those achieved with microgram-level protein subunit vaccines with adjuvants [16]. Importantly, these antibodies were capable of neutralizing multiple SARS-CoV-2 variants, including Delta and Omicron, demonstrating cross-reactive potential [16]. ELISA assays confirmed high levels of antigen-specific IgG1 and IgG2a subclasses, indicating both Th1- and Th2-skewed responses, which are critical for comprehensive antiviral immunity [47]. Neutralization assays using pseudovirus and live-virus systems consistently showed that sera from StealthX-immunized animals effectively blocked viral entry into host cells [34]. Similar results were observed for influenza H3 and RSV antigens, with multivalent formulations eliciting antibodies capable of recognizing each individual antigen without evidence of antigenic interference [17]. Most importantly, exosome based vaccine would give protection of murine model for up to 6 months with high level of plasma IgG and a boost at 6 months would immediately bring the IgG level to the original boost level (day 35). The high efficiency of humoral induction is attributed to the presentation of antigens in their native conformations on the exosome surface, which facilitates B-cell receptor engagement and subsequent germinal center reactions. Additionally, the exosome membrane may contribute adjuvant-like effects by delivering pathogen-associated molecular patterns (PAMPs) from the donor cells, further enhancing B-cell activation [48].

### 4.3. Cellular Immune Responses

Beyond antibody production, StealthX vaccines elicit robust cellular immunity, which is critical for long-term protection and clearance of infected cells. Flow cytometry and ELISPOT analyses demonstrated activation of both CD4+ helper T cells and CD8+ cytotoxic T lymphocytes in response to antigenic stimulation [35,36,49]. CD4+ T cells exhibited a Th1-biased cytokine profile, producing interferon-gamma (IFNγ), tumor necrosis factor-alpha (TNFα), and interleukin-2 (IL-2), essential for supporting cytotoxic responses and B-cell maturation [15,47]. CD8+ T cells displayed potent cytolytic activity, capable of recognizing and eliminating infected target cells. Interestingly, cellular responses were maintained across different variants of SARS-CoV-2, suggesting that StealthX-mediated antigen presentation promotes recognition of conserved epitopes [50]. Nucleocapsid-specific T-cell responses were particularly strong, complementing spike-directed neutralizing antibodies and providing an additional layer of protection against variant escape [15,51].

### 4.4. Low Dosage and Multivalent Immunity

The inclusion of multiple antigens in StealthX exosomes enhances breadth of immunity. In multivalent formulations combining spike and nucleocapsid proteins, immune responses were effectively elicited against both antigens [15]. Serum neutralization assays confirmed activity against Delta, Omicron, and ancestral SARS-CoV-2 strains, highlighting the platform’s potential for broad-spectrum protection [16,52]. For influenza H3 and RSV antigens, co-administration in a single exosome preparation did not diminish immune responses to individual antigens, confirming the absence of immune interference [17,53]. This supports the feasibility of “mix-and-match” strategies, in which exosome formulations can be rapidly adapted to emerging viral strains or combined for pandemic preparedness.

A unique advantage of StealthX is its effectiveness at nanogram-level doses, significantly lower than conventional protein subunit vaccines [15,16]. Dose-ranging studies indicated that as little as 10–50 ng of exosome-displayed antigen was sufficient to elicit high-titer antibody responses and robust T-cell activation [15,16]. Increasing the dose did not significantly improve immune responses, suggesting that exosome-mediated antigen delivery maximizes immunogenic efficiency at an extremely low dosage. This low-dose requirement has important implications for manufacturing scalability and global vaccine distribution, as it reduces the amount of antigen needed per dose, lowers production costs, and minimizes potential reactogenicity. Comparing with LNP-mRNA vaccines, StealthX offers a great potential for multiplexing different antigens (one type of antigen per exosome as shown in Figure 1) due to the mRNA dosage limitation [8]. In addition, multiplex with more mRNA in LNP-mRNA vaccines increase the risk for innate immunity activation [8]. Most importantly, StealthX vaccine demonstrated great cross-reactive and multivalent protection without immune interference at nanogram level antigen doses, which offers scalability and safety advantages [54]. These findings underscore the platform’s potential as a versatile next-generation vaccine delivery system capable of addressing current and emerging infectious disease threats.

## 5. Multivalent and “Mix-and-Match” Approaches

### 5.1. Rationale for Multivalent Vaccine Design

Viral evolution and antigenic drift pose major challenges for vaccine durability and breadth [1,50]. Platforms that can present multiple antigens (Figure 1) simultaneously are critical for maintaining protection against rapidly mutating pathogens such as SARS-CoV-2, influenza, and RSV [1]. The StealthX exosome-based vaccine platform is particularly well suited for multivalent vaccine design due to its modular architecture. Exosomes can be engineered to display diverse surface proteins or encapsulate distinct antigens within the same vesicle, enabling coordinated immune presentation without compromising antigen integrity. Traditional vaccine platforms face significant hurdles in achieving multivalency. Protein subunit vaccines require precise formulation to prevent antigen competition or structural instability [3]. Also with the dosage limitation for protein and mRNA based vaccine, multiplex would require more input, which would increase reactogenicity and excessive activation of innate immunity [8]. In addition, mRNA vaccines face delivery limitations when encoding multiple large genes. In contrast, StealthX allows direct co-display of multiple viral proteins in their native conformations (Figure 1) on a biocompatible membrane surface, promoting balanced immune activation.

### 5.2. Implementation of Multivalent StealthX Vaccines

Our papers [15,16,17] demonstrate the implementation of multivalent StealthX formulations containing combinations of SARS-CoV-2 spike and nucleocapsid proteins [16,17]. These formulations retained high yield, structural stability, and antigenic fidelity during production. Immunization with these dual-antigen StealthX exosomes elicited strong antibody and T-cell responses to both spike and nucleocapsid antigens, suggesting that antigen co-display did not cause immune interference [15]. This co-display approach addresses one of the main limitations of spike-only vaccines: rapid immune escape due to spike mutations [55]. Nucleocapsid (N) is a more conserved viral protein that contributes to durable T-cell immunity [51]. By combining S and N within the same exosome, StealthX induces both neutralizing antibodies (via S) and broad T-cell responses (via N), providing complementary layers of protection [15,55]. This strategy enhances vaccine resilience against emerging variants and may extend protection duration.

### 5.3. Mix-and-Match Flexibility

Another advantage of the StealthX platform is its mix-and-match flexibility, allowing modular integration with other vaccine technologies or sequential dosing regimens. Exosome-based vaccines can serve as either prime or boost components in heterologous vaccination schemes [56,57]. For example, a StealthX boost following a traditional mRNA or protein vaccine prime could reinforce immunity by stimulating both mucosal and systemic responses, as exosomes efficiently target antigen-presenting cells (APCs) such as dendritic cells. Conversely, a StealthX prime followed by an mRNA boost may elicit stronger memory T-cell responses due to early cross-presentation. These strategies could be tailored to specific pathogens or population needs, improving adaptability in outbreak scenarios. NCT07095231, a clinical trial originated from StealthX vaccine, running by NIAID, might provide some insight by the end of the year [58]. It would be desirable to see some clinical data to support of this idea. The ability to rapidly swap or add antigens further distinguishes StealthX from fixed-structure LNP platforms. Should a new SARS-CoV-2 variant or influenza strain arise, new antigens can be incorporated into exosomes by modifying donor cells without redesigning the entire manufacturing workflow. This plug-and-play modularity provides a practical path to rapid vaccine updates and seasonal multivalent combinations.

### 5.4. Comparative Immunogenicity of Multivalent Formulations

Preclinical results indicate that multivalent StealthX vaccines preserve or even enhance immune potency compared to monovalent counterparts. Dual-antigen exosomes containing both spike and nucleocapsid proteins generated comparable anti-spike IgG titers to spike-only formulations, while also inducing strong anti-nucleocapsid responses [15]. In T-cell assays, multivalent vaccines yielded broader cytokine profiles and greater frequencies of IFNγ–producing CD8+ cells. In comparison, mixed formulations of recombinant proteins or mRNA vaccines encoding multiple antigens sometimes exhibit antigenic interference, where immune responses to one antigen dominate at the expense of others [53]. Exosomes circumvent this issue by providing spatial separation and native presentation of each antigen within a lipid bilayer, facilitating balanced immune activation. This unique property supports StealthX’s promise as a platform for complex multivalent vaccines targeting multiple pathogens simultaneously.

### 5.5. Cross-Variant and Cross-Pathogen Protection

One of the most notable findings from the ASM Spectrum study was the demonstration of cross-variant neutralization [15]. StealthX formulations induced antibodies capable of neutralizing SARS-CoV-2 Delta and Omicron variants despite significant spike mutations [16,59]. This suggests that exosome-mediated antigen presentation elicits recognition of conformationally conserved epitopes less susceptible to mutation. Furthermore, studies incorporating antigens from influenza and RSV demonstrated that StealthX can effectively serve as a cross-pathogen vaccine platform [17]. The ability to co-package unrelated viral proteins in a single exosome formulation opens the door to pan-respiratory vaccines, potentially protecting against multiple viruses with overlapping epidemiological profiles. This is particularly relevant for vulnerable populations, where combined influenza–RSV–coronavirus vaccination could streamline immunization schedules and improve compliance [60].

### 5.6. Manufacturing and Quality Control Considerations

From a bioprocessing perspective, multivalent StealthX vaccines benefit from the scalability and flexibility of exosome manufacturing systems. Producer cells can be genetically engineered to express single antigen per cell and then a multiplexed vaccine is generated with a cocktail of exosomes (each exosome will display a different antigen) generated. Because exosomes are naturally secreted vesicles, purification can be achieved through established tangential flow filtration (TFF) and size exclusion chromatography (SEC) methods [43,44]. Characterization of multivalent exosomes involves advanced analytical techniques, including nanoparticle tracking analysis (NTA), flow cytometry, cryo-electron microscopy, and mass spectrometry [40]. These ensure consistent particle size, antigen density, and purity.

### 5.7. Broader Applications and Future Prospects

Beyond respiratory viruses, the multivalent potential of StealthX extends to other infectious diseases and oncology. For pathogens like HIV, dengue, or malaria—where antigen diversity is a major obstacle—exosomes could display conserved epitopes from multiple strains or life-cycle stages. In oncology, exosome-based formulations could incorporate tumor-associated antigens alongside immune-stimulatory molecules to generate potent anti-tumor responses [61,62]. The modularity of StealthX also supports integration with personalized medicine approaches, such as patient-specific neoantigen vaccines [63]. Because exosomes can be derived from autologous cells, they inherently minimize the risk of immune rejection and can be engineered to target specific tissues or immune cells.

### 5.8. Summary of Multivalent Advantages

The StealthX platform’s multivalent and mix-and-match (Figure 1) capabilities confer several unique advantages over conventional vaccine systems:

- Antigenic breadth: Enables co-display of multiple viral proteins, enhancing cross-protection.
- Immune balance: Promotes coordinated humoral and cellular responses without antigen interference.
- Rapid adaptability: Facilitates quick updates for emerging variants or pathogens.
- Manufacturing flexibility: Compatible with scalable bioprocesses and modular design.
- Cross-platform synergy: Supports heterologous prime-boost strategies with mRNA or protein vaccines [56,57].

These features make StealthX a leading candidate for next-generation multivalent vaccines with applications beyond COVID-19, extending to influenza, RSV, and potentially other infectious or oncologic indications.

## 6. Safety, Stability, and Translational Considerations

### 6.1. Preclinical Safety Profile

Safety is a central concern in vaccine development, and one of the defining advantages of the StealthX exosome-based vaccine platform lies in its excellent safety profile demonstrated across multiple preclinical studies. Unlike lipid nanoparticles (LNPs), which can trigger innate immune activation and inflammatory responses through ionizable lipid components [8], exosomes are endogenous vesicles naturally secreted by cells and well tolerated in vivo [13]. This biocompatibility makes them inherently less reactogenic. In murine models, repeated intramuscular administration of StealthX formulations containing nanogram quantities of antigen did not elicit any observable adverse effects. Mice exhibited no weight loss, local reactogenicity, or changes in serum biomarkers of inflammation (e.g., C-reactive protein, TNFα, or IL-6). Histopathological analyses of key organs—including liver, spleen, kidney, and lung—revealed no evidence of tissue toxicity or immune infiltration. Similar results were reported in rabbit models, supporting the cross-species safety of StealthX. Moreover, cytokine profiling indicated that StealthX induces a balanced immune activation, avoiding the excessive proinflammatory cascades often associated with adjuvanted protein or viral vector vaccines. This Th1-skewed but controlled cytokine response is particularly desirable for minimizing adverse events such as fever, myalgia, or systemic inflammation.

### 6.2. Absence of Exogenous Adjuvants

Another contributor to StealthX’s safety profile is its adjuvant-free design. Traditional protein-based vaccines require adjuvants like aluminum hydroxide or saponin derivatives to elicit sufficient immune responses, but these agents can cause injection site pain, swelling, and, in rare cases, systemic adverse events [18,64]. The StealthX platform circumvents this requirement by leveraging the immunostimulatory nature of exosome membranes themselves. Exosomes express endogenous lipids, proteins, and glycans that interact with pattern recognition receptors (PRRs) on dendritic cells and macrophages, leading to mild but effective immune activation [65]. This intrinsic adjuvanticity, coupled with the high-density display of antigens in native conformations, results in strong immune priming without the need for external adjuvants. Consequently, StealthX formulations minimize formulation complexity and reduce the risk of adverse reactions.

### 6.3. Biodistribution and Clearance

Understanding biodistribution is essential for assessing the safety of any nanoparticle-based delivery system. In contrast to synthetic nanoparticles, exosomes possess natural tropism and clearance pathways. Fluorescent and radiolabeled tracking studies have shown that intramuscularly administered StealthX exosomes primarily remain localized at the injection site for the first 24–48 h, where they are taken up by antigen-presenting cells (APCs) such as dendritic cells and macrophages [66,67]. A portion of exosomes enters local lymphatic drainage and traffics to draining lymph nodes, the principal sites of immune activation. Minimal systemic dissemination is observed, and exosomes are ultimately cleared via hepatic and renal pathways [68]. Importantly, no accumulation in off-target organs or long-term persistence has been detected, suggesting a self-limiting biodistribution profile consistent with physiological vesicle turnover. These biodistribution characteristics underscore StealthX’s biological safety advantage over synthetic nanocarriers, which may persist or accumulate in the liver or spleen, raising toxicity concerns [69]. The natural clearance kinetics of exosomes reduce the risk of chronic inflammation or tissue damage, even with repeated dosing.

### 6.4. Stability and Cold-Chain Considerations

A significant logistical advantage of the StealthX platform is its stability. Unlike mRNA-LNP vaccines, which require ultra-cold storage (−20 °C to −80 °C) to prevent RNA degradation and LNP fusion [6], exosome formulations have demonstrated stability under standard refrigeration (2–8 °C) for extended periods. The exosome’s lipid bilayer naturally protects its cargo—both surface proteins and any internal components—from enzymatic degradation. Preclinical stability studies for StealthX showed no loss of antigenic integrity or immunogenic potency after storage at 4 °C [15,16,17]. This stability profile dramatically simplifies vaccine distribution, making StealthX a more viable platform for global immunization campaigns, particularly in resource-limited settings that lack ultra-cold chain infrastructure.

## 7. Challenges, Limitations, and Future Directions

### 7.1. Overview

While the StealthX exosome-based vaccine platform demonstrates compelling preclinical promise—combining strong immunogenicity, safety, and stability—its successful translation to human use requires addressing several technical, biological, and regulatory challenges [70]. These range from ensuring large-scale manufacturing consistency to understanding immune interactions unique to exosome-derived particles. As with any emerging biotechnology, a balanced evaluation of limitations is essential for setting realistic expectations and guiding future innovation.

### 7.2. Manufacturing and Scale-Up Challenges

One of the foremost challenges for StealthX lies in scaling exosome production to clinical and commercial levels. Unlike synthetic nanoparticle platforms that can be assembled chemically, exosome manufacturing relies on living producer cells, which introduces inherent biological variability [45]. Batch-to-batch consistency, productivity, and cell line stability must be carefully controlled. Large-scale production requires high-density cell culture systems, such as hollow-fiber bioreactors or perfusion-based platforms, capable of continuous exosome secretion. Downstream purification processes—tangential flow filtration (TFF), ultracentrifugation, or size exclusion chromatography (SEC)—must be optimized for both yield and purity while maintaining vesicle integrity [43,44]. Current yields from standard adherent cultures are relatively modest, posing cost and throughput barriers to global-scale vaccine deployment [45].

Furthermore, regulatory agencies demand stringent characterization of complex biologics. Exosomes exhibit heterogeneity in size, composition, and cargo content, all of which can influence biological function [40]. Establishing reliable potency assays that correlate exosome characteristics with immunogenic outcomes remains a key technical hurdle [62]. Advances in high-resolution proteomics, nanoparticle tracking analysis, and cryo-electron microscopy will be critical for defining release criteria and ensuring reproducibility [40].

### 7.3. Antigen Loading and Display Efficiency

The precision of antigen loading is central to vaccine efficacy. Current strategies rely on fusing antigens to exosome-targeting motifs such as tetraspanins (CD9, CD63, CD81) or other membrane anchoring peptides [39]. However, differences in antigen size, folding, or hydrophobicity can affect incorporation efficiency and surface orientation. Ensuring consistent antigen density across batches is essential for predictable immune responses. Moreover, the mechanism of antigen presentation—whether on the exosome surface or within the vesicle lumen—can modulate immune outcomes. Surface-displayed antigens primarily drive B-cell activation and neutralizing antibody production, while encapsulated antigens may favor cross-presentation and T-cell priming. Achieving an optimal balance between these compartments could further enhance the breadth and durability of immune responses. Emerging engineering tools, including CRISPR-based gene editing, synthetic promoters, and exosomal display scaffolds, are being developed to enhance control over antigen presentation. Incorporating quantitative imaging and proteomic profiling will also help fine-tune exosome vaccine design for maximal immunogenic performance.

### 7.4. Heterogeneity and Biological Complexity

Another key limitation of exosome platforms is biological heterogeneity. Even when derived from clonal cell lines, exosomes exhibit variation in size (~50–200 nm), lipid composition, and protein cargo [19]. This heterogeneity complicates characterization and may affect biodistribution or immune activation. Unlike synthetic LNPs, which are chemically uniform, exosomes represent a population of vesicles with overlapping but distinct molecular profiles. While this diversity may confer biological robustness—mimicking the complexity of natural immune signaling—it also presents regulatory and analytical challenges. The field lacks standardized reference materials or consensus assays to measure exosome potency or predict in vivo efficacy [71]. Collaborative efforts between academic consortia, biotech developers, and regulatory agencies are urgently needed to define international standards for exosome-based biologics.

### 7.5. Immunological Uncertainties and Autoimmunity Risk

Despite encouraging safety data, several immunological uncertainties remain. Exosomes possess innate immunomodulatory properties, which may vary depending on their cell source or production conditions [65]. Small changes in exosome surface molecules or residual host cell proteins could theoretically influence immune tolerance or inflammation. A critical safety concern for allogeneic (non-autologous) exosome vaccines like StealthX is alloantigenicity. Exosome naturally display major histocompatibility complex (MHC) class I and class II molecules on their surface, inherited from the parental cell 293f (FreeStyke 293-F cells from ThermoFisher, Cat# R79007). When administered to an unrelated host (allogeneic injection), these foreign MHC molecules can function as alloantigens, potentially leading to the induction of allo-specific T cell responses in vivo. While no autoimmunity or off-target effects have been reported in preclinical studies, long-term human data are still lacking. To mitigate the theoretical risks of unwanted immune recognition or alloreactivity, ongoing research focuses on using minimally immunogenic cell lines or genetically engineering the donor cells to reduce the expression of highly variable MHC molecules, ensuring that immune activation is directed primarily toward the vaccine antigen rather than the carrier. Another concern involves preexisting immunity or potential cross-reactivity. If exosome formulations share epitopes with host proteins, there is a theoretical risk of unwanted immune recognition. To mitigate this, ongoing research focuses on using minimally immunogenic cell lines and human-compatible exosome scaffolds, ensuring that immune activation is directed primarily toward the vaccine antigen rather than the carrier. Additionally, the duration of immunity and need for boosters remain unknown. Exosomes may induce long-lived T-cell memory due to efficient antigen cross-presentation, but direct evidence from clinical studies is still pending [58]. Understanding the kinetics of immune persistence will be crucial for establishing optimal dosing regimens.

### 7.6. Cost, Infrastructure, and Global Access

While exosomes can be produced using scalable bioreactor systems, the cost of cell culture media, purification equipment, and quality control analytics currently exceeds that of mRNA or protein subunit platforms [45]. For StealthX to achieve global accessibility, production workflows must be streamlined and raw material costs reduced. One promising direction is the use of cell-free exosome mimetics—synthetic vesicles assembled from natural lipids but retaining exosomal surface proteins [72]. These mimetics can replicate many functional advantages of natural exosomes while simplifying production. Hybrid systems that blend natural exosome membranes with synthetic lipid components may also balance cost, scalability, and immunogenicity. In addition, regional manufacturing hubs using standardized cell banks could facilitate decentralized production, particularly in low- and middle-income countries. The platform’s stability at refrigerator temperatures (4 °C) already provides a substantial logistical advantage, but reducing manufacturing costs will be equally vital for equitable deployment.

### 7.7. Future Directions

The next generation of StealthX and related platforms will likely integrate precision targeting and modular antigen design. Functionalizing exosomes with targeting ligands—such as antibodies, peptides, or glycan modifications—could enhance delivery to specific immune compartments (e.g., dendritic cells or lymph node stromal cells) [27,73,74]. Similarly, leveraging exosomal RNA content could allow co-delivery of immunomodulatory molecules, bridging the gap between mRNA and protein-based vaccines. Machine learning and systems biology approaches are expected to play growing roles in exosome optimization, helping to predict how changes in vesicle composition influence immune outcomes. Parallel advances in manufacturing automation, analytics, and standardization will further streamline the path toward large-scale clinical implementation. Looking ahead, StealthX could evolve into a universal vaccine delivery backbone, supporting not only infectious disease prevention but also therapeutic vaccination in oncology and chronic viral infections [61,75]. Its inherent safety, adaptability, and cold-chain independence align with global public health priorities for equitable and sustainable immunization.

## 8. Conclusions

The development of StealthX, an exosome-based vaccine delivery platform, represents a significant evolution in the field of vaccinology. By leveraging the natural intercellular communication properties of exosomes, StealthX bridges the gap between synthetic and biological delivery systems. It offers a unique combination of safety, stability, and precision immunogenicity unmatched by current mRNA–LNP or protein-based platforms [10,15]. Unlike lipid nanoparticles, which may trigger innate inflammatory pathways or require ultra-cold storage [6,8], exosomes present antigens in a biomimetic context that closely resembles physiological immune signaling, ensuring efficient cellular uptake with minimal toxicity. Through rational design, StealthX overcomes traditional barriers in vaccine development—enhancing antigen presentation, supporting both humoral and cellular immune responses, and enabling flexible antigen customization. Currently, a Phase I clinical trial is running by NIAID for safety evaluation [58]. Nonetheless, the platform’s long-term clinical translation requires resolution of key challenges in manufacturing scalability, product characterization, and regulatory standardization [45,71]. The inherent complexity of biologically derived particles necessitates rigorous analytical and process control frameworks to ensure reproducibility and regulatory compliance. Progress in cell engineering, exosome analytics, and bioprocess optimization will be pivotal to unlocking StealthX’s full potential. Looking forward, the convergence of exosome biology with synthetic biology, machine learning, and modular vaccine design could redefine how immune responses are programmed. In sum, StealthX exemplifies the next generation of vaccine technology—not just a biologically inspired, versatile, and globally scalable platform, but also holds great promise in oncology, personalized medicine, and therapeutics immune modulation [61,63,76].

Looking ahead, continued advances in exosome engineering, scalable manufacturing, and standardized analytical frameworks will be critical to unlocking the full clinical potential of the StealthX platform. The outcome of ongoing and future human studies will ultimately determine the durability, breadth, and translational value of exosome-based vaccines. Beyond infectious diseases, the modularity and targeting flexibility of StealthX also position it as a promising backbone for therapeutic vaccination in oncology and for personalized immunotherapy applications.

## Figures and Tables

**Table 1:** Common Methods and Assays for Exosome Characterization.

Category	Assay	Description
Particle Size and Concentration	Nanoparticle Tracking Analysis (NTA)	Tracks Brownian motion of particles in liquid to determine size distribution and concentration (~50–200 nm typical for exosomes).
	Dynamic Light Scattering (DLS)	Measures fluctuations in light scattering to estimate average particle size and polydispersity; less precise for heterogeneous samples.
	Tunable Resistive Pulse Sensing (TRPS)	Measures electrical resistance changes as particles pass through a nanopore, providing accurate size and concentration distribution.
Morphology and Structure	Transmission Electron Microscopy (TEM)	Visualizes exosome morphology at nanometer resolution; confirms cup-shaped vesicles.
	Cryo-Electron Microscopy (Cryo-EM)	Preserves vesicle structure in near-native hydrated state for more accurate morphology assessment.
	Atomic Force Microscopy (AFM)	Scans surface topology and mechanical properties of individual exosomes on a substrate.
Marker Protein Identification	Western blot (WB)	Detects key exosomal markers (e.g., CD9, CD63, CD81, TSG101, Alix) and negative markers (e.g., Calnexin).
	Flow Cytometry (Conventional or Nano-FACS)	Measures surface protein expression on single exosomes or bead-coupled exosomes using fluorescent antibodies.
	ELISA (Enzyme-Linked Immunosorbent Assay)	Quantifies specific exosomal proteins in bulk samples.
	Mass Spectrometry (Proteomics)	Provides a global proteome profile of exosomal proteins for biomarker discovery.
Nucleic Acid Content	qPCR/RT-qPCR	Detects and quantifies exosomal RNA (miRNA, mRNA, lncRNA).
	RNA-seq	Provides comprehensive profiling of RNA species within exosomes.
	Bioanalyzer or TapeStation	Determines RNA size distribution and integrity (typically short RNAs for exosomes).
Purity and Composition	Protein-to-Particle Ratio (via BCA + NTA)	Compares total protein to vesicle count; higher ratio suggests protein contamination.
	Density Gradient Ultracentrifugation	Separates exosomes from protein aggregates and other EVs based on buoyant density (1.13–1.19 g/mL typical).
	Immunoaffinity Capture	Uses antibodies to isolate vesicles expressing specific exosomal markers for purity assessment.
Functional and Uptake Studies	Cell Uptake Assay (Fluorescent Labeling)	Labels exosomes (e.g., PKH26, DiO) to track their internalization by recipient cells using fluorescence microscopy or flow cytometry.
	Reporter Assays	Uses reporter genes or fluorescent cargo to confirm functional delivery of exosomal contents.
	In Vitro Functional Assays	Measures biological outcomes such as proliferation, migration, or immune modulation in target cells after exosome treatment.

## Data Availability

The original contributions presented in this study are included in the article. Further inquiries can be directed to the corresponding author.
